# Noradrenaline has opposing effects on the hydraulic conductance of arterial intima and media

**DOI:** 10.1016/j.jbiomech.2017.01.027

**Published:** 2017-03-21

**Authors:** K.Y. Chooi, A. Comerford, S.J. Sherwin, P.D. Weinberg

**Affiliations:** a Department of Bioengineering, Imperial College London, United Kingdom; b Department of Aeronautics, Imperial College London, United Kingdom

**Keywords:** Permeability, Filtration, Barrier, Artery, Vasoconstriction, Norepinephrine, Noradrenaline, Atherosclerosis

## Abstract

The uptake of circulating macromolecules by the arterial intima is thought to be a key step in atherogenesis. Such transport is dominantly advective, so elucidating the mechanisms of water transport is important. The relation between vasoactive agents and water transport in the arterial wall is incompletely understood. Here we applied our recently-developed combination of computational and experimental methods to investigate the effects of noradrenaline (NA) on hydraulic conductance of the wall ($L_{p}$), medial extracellular matrix volume fraction ($\phi^{\mathrm{ECM}}$) and medial permeability ($K_{1}^{1}$) in the rat abdominal aorta. Experimentally, we found that physiological NA concentrations were sufficient to induce SMC contraction and produced significant decreases in $L_{p}$ and increases in $\phi^{\mathrm{ECM}}$. Simulation results based on 3D confocal images of the extracellular volume showed a corresponding increase in $K_{1}^{1}$, attributed to the opening of the ECM. Conversion of permeabilities to layer-specific resistances revealed that although the total wall resistance increased, medial resistance decreased, suggesting an increase in intimal resistance upon application of NA.

## 1 Introduction

The uptake of lipid-carrying plasma macromolecules by the arterial wall is thought to be a critical factor in the development of atherosclerosis (Weinberg, 2004, Tarbell, 2003, Tarbell, 2010). The characteristically patchy accumulation of such macromolecules is the result of complex transport mechanisms into and within the arterial wall which are only partially understood. Given that such macromolecular transport is dominantly advective (Tedgui and Lever, 1985), elucidating the mechanisms of water transport is a key step towards understanding macromolecule accumulation. Our previous studies have demonstrated that medial hydraulic resistance accounts for most of the total wall hydraulic resistance within the physiological pressure range, even in the relatively thin-walled rat aortic bifurcation (Chooi et al., 2016). The medial permeability to water in atheroprone arteries is therefore of interest.

The medial layer of the arterial wall consists of vascular smooth muscle cells (SMCs) surrounded by a complex network of elastin, collagen, proteoglycans and glycosaminoglycans. Changes to the structure of this layer are likely to have an impact on the transport of water and solutes across the wall. Our previous study (Chooi et al., 2016) investigated influences of changes in structure resulting from alteration of transmural pressure. It was found that the structural rearrangement of the solid components of the media gives rise to a nonlinear relation between permeability and wall strain. However, there is also an active mechanical mechanism – SMC contraction – that could alter medial (and hence wall) permeability through effects on structure.

When stimulated, SMCs alter their tone or actually contract (i.e. shorten along their long axis) depending on the transmural pressure gradient and hence stretch of the wall (Zulliger et al., 2002); under isobaric conditions, the luminal diameter decreases and wall thickness increases (Rachev and Hayashi, 1999). SMC contraction can be induced by the nervous system, by chemical signals transported in the blood and by locally-released paracrine mediators (Ludmer et al., 1986). Hypertension and obesity are examples of systemic conditions associated with increased SMC tone (Fridez et al., 2001, Meyer et al., 2013); both are important risk factors for atherosclerosis, and may act at least in part by influencing medial transport properties. Examples of paracrine mediators are the endothelium-derived constrictor endothelin (ET-1) and dilator nitric oxide (NO) (Bourque et al., 2011). More recently, a role of perivascular adipose tissue in SMC tone control has been reported; it acts via another set of vasoactive molecules yet to be identified (Meyer et al., 2013). An important implication of these sources of paracrine signalling is the potential existence of heterogeneous distributions of vasodilators and vasoconstrictors within the tunica media, leading to spatially varying medial permeability. This could account in part for the patchy distribution of macromolecular accumulation and atherosclerosis.

Here, we have applied our combined numerical/experimental method (Comerford et al., 2015) to investigate the effects of noradrenaline (NA) on water transport properties of the whole arterial wall and its component layers. Effects on transmural water flux were obtained by direct measurement, effects on medial permeability were obtained by numerical methods using experimentally-derived boundary conditions, and intimal hydraulic resistance was obtained by subtraction.

## 2 Methods

### 2.1 Overview

**Fig. 1:**
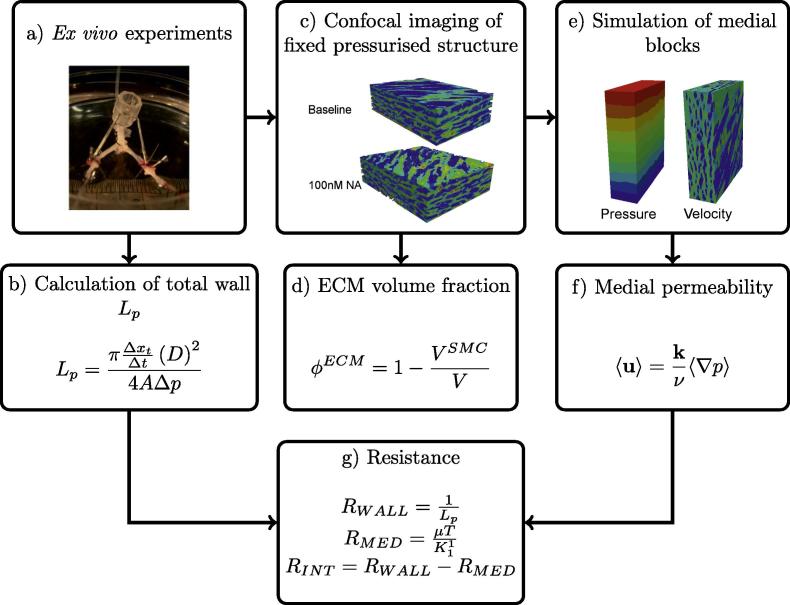
Flowchart describing major steps in the combined computational/experimental method.

The effect of NA-induced vasoconstriction on arterial wall hydraulic conductance, $L_{p}$, was investigated using an *ex vivo* preparation of the rat aortic bifurcation (Fig. 1(a) and (b)) described previously (Chooi et al., 2016). The aortic bifurcation is a common site for atherosclerosis (Mitchell and Schwartz, 1965); stenosis at this location is a major cause of peripheral arterial disease. To distinguish between SMCs and extracellular matrix (ECM) of the wall, and hence to provide the microstructure for the numerical simulations of medial transport, bovine serum albumin (BSA) labelled with the fluorescent dye Lissamine™ rhodamine (Rh-BSA), was added to the luminal fluid and its transport was allowed to reach a steady state across the arterial wall. Following completion of the $L_{p}$ measurements, the Rh-BSA was chemically fixed by perfusion at pressure and its distribution was imaged by confocal microscopy (Fig. 1(c)). Image volumes were transformed onto a structured computational grid and SMCs and other areas inaccessible to the albumin tracer were removed from the domain using a penalty parameter,^1^ effectively treating the SMCs and fibres with pores sufficiently small to exclude albumin as impermeable objects. This gave realistic geometries for flow simulations.

Flow was simulated in medial tissue blocks driven by pressure gradients imposed in each of the three orthogonal axes and the intrinsic permeability was calculated (Fig. 1(e) and (f)). The permeability of the ECM was assumed to remain unchanged under the influence of NA; the implications of this assumption are discussed below. The ECM volume fraction was also quantified in each medial block (Fig. 1(d)). Medial thickness was measured from confocal images that were rotated and aligned with the radial direction. Finally, the total wall hydraulic resistance was decomposed into medial and intimal components by subtracting the computationally-obtained medial resistance from the experimentally-measured whole wall resistance, thus elucidating the effects of NA on medial and intimal hydraulic resistance (Fig. 1(g)).

### 2.2 Animals

All animal procedures were approved by the Local Ethical Review Panel of Imperial College London and complied with the Animals (Scientific Procedures) Act 1986. Eight male Sprague Dawley rats (271.5 ± 6.5 g; mean ± SEM; Charles River, UK) were fed a normal laboratory diet (LBS Biotechnology Ltd, UK) *ad libitum* and housed under a 12 h light cycle at 20–25 °C.

### 2.3 Vessel isolation

The *ex vivo* methods used in this study were based on previous work, described in Chooi et al. (2016). Briefly, animals were anaesthetised with isoflurane and the distal abdominal aorta and proximal iliac arteries were cannulated and removed. A system of reservoirs provided a constant hydrostatic pressure (Tedgui and Lever, 1984, Forster and Weinberg, 1997) and prevented collapse or over-pressurisation of the arteries during the isolation. The cannulae were tied to a stereotactic tripod before removal of the vessels from the body to maintain arterial segment lengths and the bifurcation angle at their *in vivo* values. The entire preparation was placed into a temperature-controlled bath of Tyrode’s Salt Solution (TSS; composition in g/l was 8 NaCl, 0.2 KCl, 0.2 CaCl_2_, 0.1 MgCl_2_, 0.05 NaH_2_PO_4_, 1 NaHCO_3_, 1 glucose; pH 6.5) at 37 °C that had been pre-equilibrated with 95% air and 5% CO_2_.

**Fig. 2:**
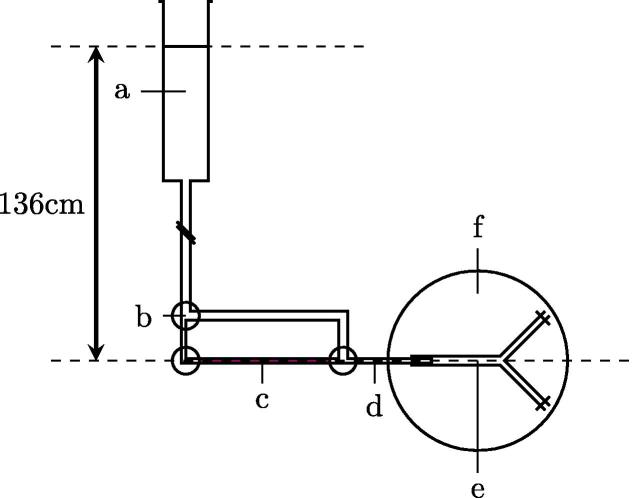
Diagram of *ex vivo* vessel perfusion. (a) TSS reservoir above the vessel, (b) 3-way tap, (c) tracer solution, (d) graduated capillary: Inner diameter = 460μm, length = 30cm, (e) isolated aortic bifurcation: Aortic length = 11±0.5mm, Iliac length = 8±0.5mm, (f) temperature-controlled abluminal bath. Adapted from Chooi et al. (2016).

Fig. 2 shows the system used to perfuse the vessel at pressure *ex vivo*. TSS supplemented with 1% Rh-BSA and 3% unlabelled BSA was introduced into the lumen and the abluminal TSS was replaced with TSS containing 4% unlabelled BSA.

### 2.4 Hydraulic conductance experiments

Steady state $L_{p}$ was measured in arteries exposed to an increasing concentration of NA using methods described previously (Chooi et al., 2016). Baseline $L_{p}$ in the absence of NA was measured in each specimen. NA concentration in the abluminal bath was then increased stepwise (1 nM, 100 nM, 10 μM), allowing water transport to reach steady state after each increase in concentration before re-assessing $L_{p}$.

### 2.5 Microscopy and image processing

#### 2.5.1 Fixation of arteries at pressure and embedding

Steady state tracer distributions were obtained after completion of $L_{p}$ measurements. Vessels were placed into a fresh abluminal saline bath containing 100 nM NA until steady state transmural flux of tracer was reached. Fixation and dehydration followed immediately as described by Chooi et al. (2016). The deformation induced by the 100 mmHg transmural pressure and the original vessel lengths and angles were maintained by performing the fixation without removing the vessel from the stereotactic and perfusion apparatus. The use of formal sublimate (6% HgCl_2_ in 15% formaldehyde) prevented elastic recoil of the vessel when it was released from the apparatus; our previous study (Chooi et al., 2016) showed that preserved length was ∼100% of the original vessel length with this fixative but not with formaldehyde on its own.

#### 2.5.2 Confocal microscopy

The lateral walls were imaged in 3D at a position 2 mm proximal to the apex of the bifurcation. (For full details, see Comerford et al. (2015).) Briefly, embedded arteries were cut in the frontal plane so that the cut face showed a longitudinal section. The cut face was imaged using an inverted laser scanning confocal microscope (Leica, TCS SP5) with the *z*-axis of the *z*-stack aligned perpendicularly to the cut face. Rhodamine fluorescence was excited at 575 nm; emission was imaged at 585–595 nm.

#### 2.5.3 Image processing

**Fig. 3:**
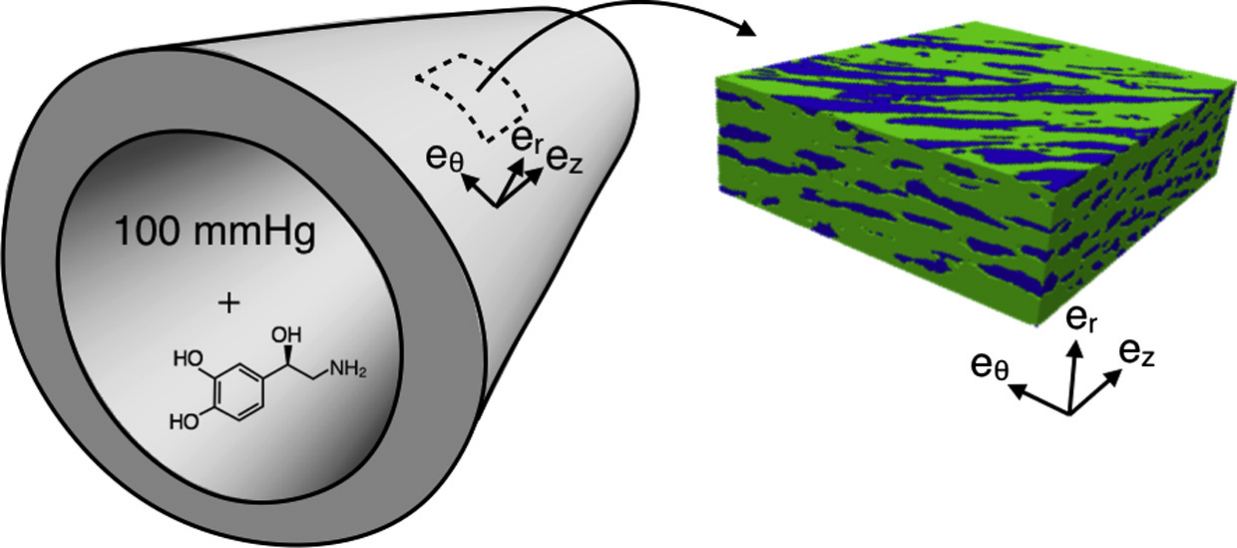
Extraction of a representative region of medial tissue from a 3D confocal image of the fixed artery. The arterial geometry on the left shows the cylindrical coordinate system relative to the artery of an example location for tissue extraction. The medial block on the right shows how the cylindrical coordinates of the artery are represented relative to the tissue block. $e_{r}\text{,}e_{\theta }$ and $e_{z}$ are the radial, circumferential and axial directions, respectively.

Five cuboidal blocks were extracted from images of three pieces of tissue from the baseline group. A further four blocks were extracted from images of three pieces of tissue fixed at 100 nM NA. An example and coordinate orientation of a block is shown in Fig. 3. A correction for intensity attenuation with depth was performed using Fiji (Schindelin et al., 2012) as described previously (Comerford et al., 2015) and three image volume rotations were applied to align the imaging axes to the cylindrical coordinates of the aorta. Medial thickness was measured after image rotations were applied.

### 2.6 Effective permeability

To determine the effective permeability of a porous medium, the flow field must be determined. Flow around solid objects embedded in a porous matrix is described by Brinkman’s equation (see Wang and Tarbell, 1995, Huang and Tarbell, 1997, Comerford et al., 2015). In the arterial media the solid objects are the SMCs and impervious fibrous proteins, and the surrounding medium is the porous ECM. The chosen isotropic value for ECM permeability, $k_{\mathrm{ECM}}=1.32\times 10^{-18}$ m^2^, was taken from the mean of published values (Wang and Tarbell, 1995, Huang and Tarbell, 1997, Dabagh et al., 2009). Although these published values were measured in rabbit tissue, ECM structure and behaviour are similar between vertebrate species (Wagenseil and Mecham, 2009).

We recently outlined an efficient approach to determine the effective permeability of the arterial media using Brinkman’s equation (Comerford et al., 2015) and implemented it in the spectral/hp element framework Nektar++ (Cantwell et al., 2015). Briefly, we first determine the flow around SMCs in a representative region of the realistic microstructure obtained from 3D confocal imaging data (Fig. 3, the green tissue represents the ECM and the blue regions the SMCs and impervious fibrous proteins). The method treats the impermeable objects by applying a penalty parameter that ensures flow travels around rather than through them. The flow field is determined in each of the main coordinate directions of each block taken from the arterial wall (coordinates shown in Fig. 3) subject to a pressure drop in that direction. From these simulations we can determine mean volumetric velocity (〈u〉) and pressure gradients (〈∇p〉) using Darcy’s law:

(1) $$\langle u\rangle =\frac{k}{\nu }\langle \nabla p\rangle \text{,}$$

where ν is the kinematic viscosity and k is the permeability tensor:

(2) $$k=\left[\begin{array}{ccc} k_{\mathrm{rr}} & k_{\mathrm{rz}} & k_{r\theta }\\ k_{\mathrm{rz}} & k_{\mathrm{zz}} & k_{z\theta }\\ k_{r\theta } & k_{z\theta } & k_{\theta \theta } \end{array}\right]$$

The volume-averaged results are then combined to form an over-determined system of equations that can be solved using a least squares approach to find the components of k. This amounts to a homogenisation of the microscale transport to provide a macroscopic description. The tensor in Eq. (2) can be diagonalised to find the principal components of fluid transport of the arterial wall ($K_{1}^{1}$ is the radial component and $K_{1}^{2}$ and $K_{1}^{3}$ are the two transverse components). We focus on the radial principal component; this is the dominant transport direction as the transmural pressure gradient has the largest magnitude.

### 2.7 ECM volume fraction

The confocal data were transformed onto the quadrature points of the computational mesh (64,000 mesh elements, $8\times 10^{6}$ quadrature points). Thresholding fluorescence intensities divided the volume into two compartments, the volume occupied by the SMC and impervious fibres and the remaining volume, corresponding to the ECM. Previously, we have found that the error in the calculated volume due to inter-observer variations in the chosen threshold value is ∼2% (Chooi et al., 2016). The volume fraction of the ECM ($\phi^{\mathrm{ECM}}$) of a medial block with volume *V* can then be defined by:

(3) $$\phi^{\mathrm{ECM}}=\frac{V^{\mathrm{ECM}}}{V}\text{.}$$

Between the baseline and 100 nM NA the increase in volumetric strain generated within the ECM can be determined. This strain relative to the baseline is given by:

(4) $$J=\frac{\phi^{\mathrm{ECM}}}{\phi_{B}^{\mathrm{ECM}}}\text{,}$$

where $\phi_{B}^{\mathrm{ECM}}$ is the volume fraction of the ECM in the baseline configuration.

### 2.8 SMC aspect ratio

The aspect ratio (AR) of SMCs in baseline and constricted conditions was calculated. SMCs were separated in each slice of each image stack using watershed segmentation in the scikit-image processing library (van der Walt et al., 2014). This segmentation algorithm allows apparently-connected SMCs to be separated into two distinct cells. In brief, the confocal images were manually thresholded to form binary images. For the binary image, the foreground represents the ECM and the background represents the SMCs. In each slice, the distance of each background pixel from the nearest foreground pixel is calculated. When this distance is plotted as a height above the image and inverted to produce a heightmap, the SMCs are visualised as a series of basins, divided by ridges. The watershed algorithm then floods the basins up to the height of the ridges. The line on which two basins meet is termed a watershed and represents the boundary of two adjacent cells. AR, defined as the minor diameter over the major diameter, was determined for each cell. The results were collected into 50 bins.

### 2.9 Intimal hydraulic resistance

Intimal hydraulic resistance, $R_{\mathrm{INT}}$ was determined by a combination of our experimental and computational results. Total wall hydraulic resistance, $R_{\mathrm{WALL}}$ was calculated from experimental measurements of $L_{p}$:

(5) $$R_{\mathrm{WALL}}=\frac{1}{L_{p}}\text{.}$$

Medial resistance, $R_{\mathrm{MED}}$, was obtained from medial permeability ($K_{1}^{1}$), medial thickness (*T*), measured from the confocal images, and the viscosity of water (μ):

(6) $$R_{\mathrm{MED}}=\frac{\mu T}{K_{1}^{1}}\text{.}$$

As the layers of the arterial wall are arranged in series, intimal resistance can be calculated as follows:

(7) $$R_{\mathrm{INT}}=R_{\mathrm{WALL}}-R_{\mathrm{MED}}\text{.}$$

## 3 Results

### 3.1 Whole wall hydraulic conductance

**Fig. 4:**
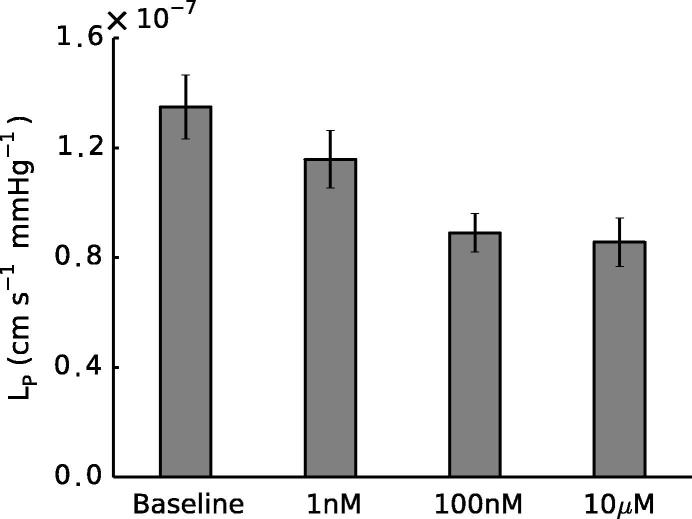
Total wall $L_{p}$ was significantly reduced in the presence of NA at concentrations ≥ 100nM (p=0.004,n=7 at 100 nM; p=0.001,n=5 at 10 μM, paired *t*-tests). Error bars represent SEM.

Measurements of $L_{p}$ showed that exposing the artery to 100 nM NA was sufficient to achieve a significantly lowered conductance (p=0.004, paired *t*-test) and that a further increase in concentration did not produce a greater response (Fig. 4). Hence all computational studies focused on two conditions: baseline (0 M NA) and 100 nM NA, henceforth referred to as the constricted condition.

### 3.2 Medial geometric measurements

**Fig. 5:**
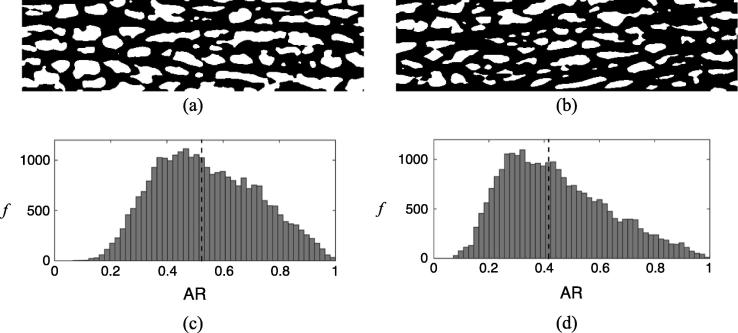
(a) & (b) Thresholded images of tracer in the media in a single *r*-*z* slice (Dimensions: 21.28×79.8μm) of the 3D confocal stack for typical baseline and constricted samples respectively: white areas represent SMCs and impervious fibres; black areas represent ECM. (c) & (d) Frequency histograms showing aspect ratio (AR) of SMCs for baseline and constricted samples respectively. The black dotted line represents the median AR.

The images shown in Fig. 5(a) and (b) are representative slices in the *r*-*z* plane of 3D images of baseline and constricted samples. The difference in SMC size is visually evident: the baseline sample contains larger, wider cells than the constricted tissue. Cells do not align circumferentially but have a helical orientation. Some SMCs appear smaller than others since the cells do not have uniform cross sectional area but rather have a fusiform shape (Clark and Glagov, 1985).

The visual observations in Fig. 5(a) and (b) are characterised quantitatively by considering a histogram of the SMC ARs (Fig. 5(c) and (d)). Between baseline and 100 nM NA there is a shift of the histogram to the left demonstrating that the SMCs have a lower AR in their contracted state (median AR values for baseline and contracted were 0.5232 and 0.4163, respectively).

Table 1 summarises $\phi^{\mathrm{ECM}}$ and medial thickness, *T*. Between baseline and 100 nM NA, $\phi^{\mathrm{ECM}}$ increased ∼12%. This also represents the change in volumetric strain between baseline and the constricted state (see Eq. (4)). *T*, measured from confocal image volumes and expressed as a combined mean of the iliac and aortic region, increased ∼20% (*p* = 0.044).

### 3.3 Medial hydraulic permeability

**Fig. 6:**
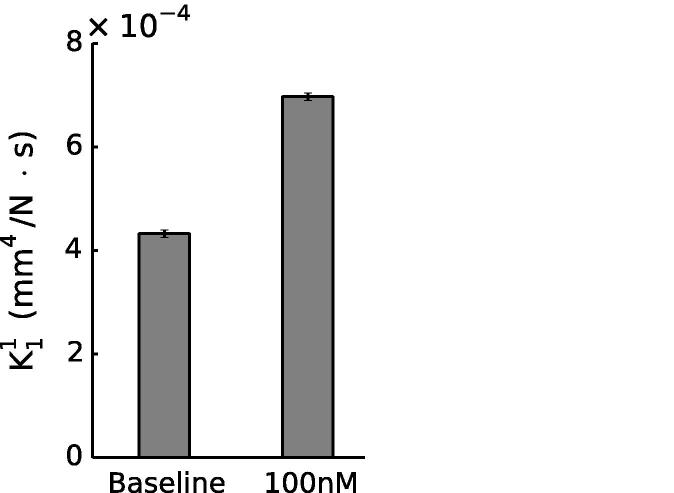
Radial medial hydraulic permeability was significantly increased in arteries constricted with 100 nM NA ($p=4.50\times 10^{-8}$, unpaired *t*-test). Error bars represent SEM. *n* = 5 (baseline); *n* = 4 (constricted).

Radial hydraulic permeabilities from numerical simulations are shown in Fig. 6. Results for individual simulations are shown in Table 2. $K_{1}^{1}$ in the constricted case was significantly higher than in baseline controls (∼61% increase, $p=4.50\times 10^{-8}$, unpaired *t*-test). $K_{1}^{2}$ and $K_{1}^{3}$ were 2.2–2.6 times greater than $K_{1}^{1}$ in controls and 1.6–1.8 times greater in the constricted state. This reflects the anisotropy of the tissue (see Comerford et al. (2015)). However, water flux is not greater in these directions as the predominant pressure drop is in the radial direction.

### 3.4 Decomposition of wall hydraulic resistance

**Fig. 7:**
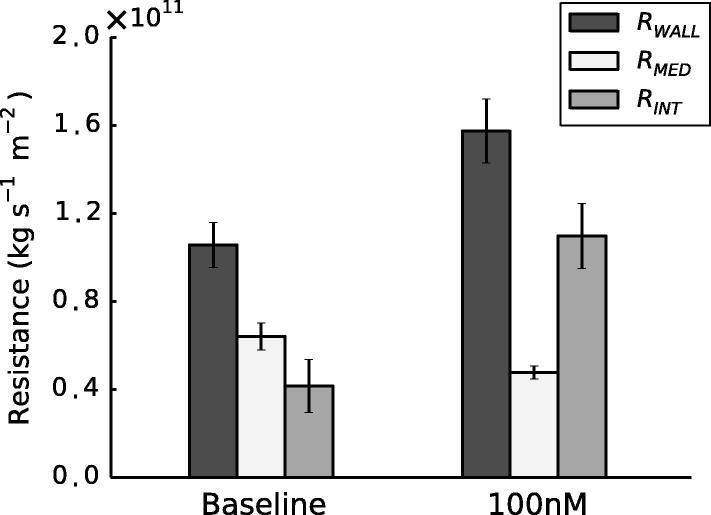
Decomposition of total wall hydraulic resistance at baseline and with 100 nM NA. Error bars represent SEM. *n* = 8 (baseline); *n* = 7 (constricted).

As described in Section 3.1, $L_{p}$ was significantly lower in constricted arteries than in baseline controls (p=0.004, paired *t*-test). Thus $R_{\mathrm{WALL}}$ was significantly higher in constricted arteries ($p=8.87\times 10^{-4}$, paired *t*-test). Paradoxically, a corresponding reduction in $R_{\mathrm{MED}}$ was observed with NA. $K_{1}^{1}$ was significantly higher in constricted samples than in baseline ($p=4.50\times 10^{-8}$) and medial thickness (*T*), measured from confocal images,^2^ increased from 27.7±2.16μm to 33.2±1.7μm. These values were used in Eq. (6). Despite a ∼20% increase in medial thickness the 61% increase in $K_{1}^{1}$ produced the overall decrease in $R_{\mathrm{MED}}$. Applying the electrical resistance analogy set out in Section 2.9, a 2.6-fold increase in $R_{\mathrm{INT}}$ was observed (Fig. 7).

## 4 Discussion

During contraction, SMCs change in shape, shortening along their long axis and widening in the other two axes (Seifter et al., 2005). The long axes of SMCs are usually aligned at some angle within the *z*-θ plane (Holzapfel et al., 2002). Previously, it has been shown that following SMC contraction the inner diameter decreases and the wall thickness increases to reduce circumferential stress (Rachev and Hayashi, 1999). In our experiments, the latter was observed, with an increase of ∼20% in *T*.

In addition to the change in medial thickness, we observed an increase in $\phi^{\mathrm{ECM}}$ (Table 1) between baseline and 100 nM NA, leading to the 61% increase in $K_{1}^{1}$ (Fig. 4). $\phi^{\mathrm{ECM}}$ expressed as *J* (see Section 2.7) showed a 12% increase in volumetric strain, which suggests an increase in the space available for water transport to occur. The dilatation of the ECM under SMC contraction is due to cell-generated forces pulling on the fibres of the ECM: contractile elements within SMCs are known to be mechanically connected to extracellular fibres (e.g. collagen) through integrins (Moiseeva, 2001, Ye et al., 2014, Bursa et al., 2011), causing a widening of intercellular channels as seen in Fig. 5 and increasing the strain in the matrix.

Contraction of SMCs was seen to change their cross-sectional shape: in the baseline configuration, the cross-section of the SMCs was more circular whilst in the constricted state, the SMCs flattened in the radial direction, evidenced by a skew of the AR towards zero.

A 100 nM NA concentration was sufficient to produce a significant decrease in $L_{p}$ (Fig. 4). Higher concentrations had no further effect. Hence the experimental data show that $R_{\mathrm{WALL}}$ increases upon NA-induced contraction. However, the medial permeability data, derived from a numerical simulation based on images of fixed tissue, showed a decrease in $R_{\mathrm{MED}}$. Although the decrease in $R_{\mathrm{MED}}$ may seem counter-intuitive, given the 20% increase in medial thickness, this is outweighed by the increase in medial permeability of 61%. $R_{\mathrm{INT}}$ must have increased with NA in order to explain the overall increase in $R_{\mathrm{WALL}}$ despite the decrease in $R_{\mathrm{MED}}$.

We are not aware of any previous studies examining effects of NA on $L_{p}$ of endothelium or intima in large vessels. Several studies have shown that NA reduces the permeability of cultured aortic or pulmonary artery endothelium to albumin or dextrans (Langeler and Van Hinsbergh, 1991, Griffin and Moorman, 1994, Zink et al., 1993, Bottaro et al., 1986); these results imply that transport through intercellular junctions is reduced and hence are consistent with the reduction of intimal hydraulic conductance that we observed. (Note, however, that transport through intercellular junctions is artefactually elevated in culture (Albelda et al., 1988) for unknown reasons and hence this agreement may be unreliable.)

A study of $L_{p}$ in individually-perfused capillaries of the frog mesentery showed no effect of NA (Huxley et al., 1992). A difference in the properties of capillary and large-vessel endothelium may explain the discrepancy between this result and our own data. An alternative explanation is that NA reduced vessel diameter in the present study but is unlikely to have done so in capillaries, which are devoid of SMC. We speculate that a reduction in diameter (and hence in endothelial surface area) could lead to buckling or thickening of the endothelium and hence could influence $L_{p}$ by making intercellular junctions longer and/or narrower. Consistent with this view, our previous study (Chooi et al., 2016) found that $L_{p}$ decreased when diameter was reduced by lowering the transmural pressure difference.

One limitation of this study is the use of the same value of $k_{\mathrm{ECM}}$ in all simulations. Although it is plausible that SMC contraction would open pores in the ECM, increasing its permeability, this effect would be small compared to that of increasing the ECM volume fraction, which was taken into account in the simulations – Table 1 and Fig. 6 show that a small change in ECM volume fraction causes a large change in radial permeability. Furthermore, we note that any increases in $k_{\mathrm{ECM}}$ produced by NA would exaggerate rather than negate the effects that we present: $R_{\mathrm{MED}}$ would be even further reduced by NA, and $R_{\mathrm{INT}}$ would consequently be further increased. As an additional check, we tested both an increase and decrease in $k_{\mathrm{ECM}}$ of 20%. An increase in $k_{\mathrm{ECM}}$ meant the contribution of the media to wall resistance was decreased to a level slightly higher than that of the intima ($R_{\mathrm{MED}}$ ∼51% of $R_{\mathrm{WALL}}$); the endothelium still dominated in the NA-induced contracted state (∼75% $R_{\mathrm{WALL}}$). A decrease in $k_{\mathrm{ECM}}$ meant the contribution of the media to wall resistance increased ($R_{\mathrm{MED}}$ ∼76% of $R_{\mathrm{WALL}}$); the endothelium represented ∼63% of the wall resistance in the NA-induced contracted state. In all cases, therefore, a consistent trend was observed.

Finally, we speculate briefly concerning the relevance of the results to atherosclerosis. NA increased the resistance of the intima to water flux but decreased the resistance of the media. If these trends also hold for the transport of large solutes, which are dominantly transported by advection, then NA might reduce their influx into the intima and increase their efflux across the media, leading to a decreased intimal accumulation. Effects of NA might be even more pronounced in muscular arteries, where we would expect larger diameter changes. Depending on whether the solute was pro- or anti-atherogenic (e.g. low- and high-density lipoproteins, respectively), this could have a beneficial or adverse effect on disease development.

## Conflict of interest

The authors declare no conflict of interest in relation to the material presented in this manuscript.

## Figures and Tables

**Table 1:** Table of geometric measurements showing mean ± SEM in baseline and constricted states: ECM volume fraction was significantly increased in the media of constricted arteries ($p=9.12\times 10^{-4}$, unpaired *t*-test). Medial thickness increased ∼20% from baseline control to constricted case (*p* = 0.044, unpaired *t*-test).

	Baseline (*n* = 5)	100 mM (*n* = 4)
$\phi^{\mathrm{ECM}}$	0.60±0.01	0.68±0.01
*T* (μm)	27.7±2.2	33.2±1.7

**Table 2:** Table of radial medial hydraulic permeability results obtained from simulations. Radial medial hydraulic permeability was significantly increased in arteries constricted with 100 nM NA ($p=4.50\times 10^{-8}$, Students unpaired *t*-test).

	Baseline (*n* = 5)	100 mM (*n* = 4)
	4.49	7.20
	4.50	6.95
	4.37	6.95
	4.11	6.79
	4.17	
		
Mean	4.33	6.97
SEM	0.0812	0.0844

## Footnotes

1 This parameter drives the permeability towards zero.

2 The thickness *T* is a mean of 20 samples taken from the aorta and iliac regions.
